# Solubility, Release Behavior and Membrane Permeability of a Ibuprofen Hydrogel Co-Assembled with N-Methyl-D-Glucosamine

**DOI:** 10.3390/gels12070577

**Published:** 2026-06-29

**Authors:** Guoxun Li, Xinru Lu, Caijuan Hu, Jiaxuan Ji, Xiakang Xiong, Yujia Zhang, Zhenwei Ni, Jue Wang, Jiawei Han, Xiaoqian Liu

**Affiliations:** School of Pharmacy & School of Biological and Food Engineering, Changzhou University, Changzhou 213164, China; liguoxun@cczu.edu.cn (G.L.); 14752109014@163.com (X.L.); hcj@cczu.edu.cn (C.H.); jjx1226zzz@163.com (J.J.); 18913431895@163.com (X.X.); yujia_zhang777@163.com (Y.Z.); nizhenwei811@163.com (Z.N.); wangjue@cczu.edu.cn (J.W.)

**Keywords:** ibuprofen, *N*-methyl-*D*-glucosamine, solubility, in vitro release, membrane permeability

## Abstract

Small-molecule hydrogels have gradually become a research hotspot compared with polymeric hydrogels, but their practical advantages have not been fully realized in the development of pharmaceutical formulations. This study aimed to explore whether the N-methyl-D-glucosamine (GLU) could be introduced to form a ibuprofen (IBU) hydrogel for overcoming its water solubility defect and optimizing its pharmaceutical properties. Such an IBU-GLU hydrogel was prepared by simply mixing IBU with GLU in small-volume deionized water. The formed IBU-GLU hydrogel was characterized by SEM, rheology, DSC, PXRD and FTIR analyses. In addition, the solubility, in vitro release and permeability were also investigated to evaluate the solubilization and permeability-promoting effects. The resulting IBU-GLU hydrogel exhibited a typical 3D structure with excellent viscoelasticity, which relied on the equilibrium of aggregation and dissolution, as well as a good miscibility between IBU and GLU, and self-assembly driven by intermolecular interactions in an aqueous environment. Compared to pure IBU, the IBU solubility of the IBU-GLU hydrogel was significantly improved by 38.4-fold. Furthermore, IBU-GLU hydrogel demonstrated superior release rates and supersaturation ability, which was attributed to its high-energy state and internal molecular complexation. Additionally, compared with the commercially available IBU hydrogel, the prepared IBU-GLU hydrogel significantly accelerated IBU membrane permeation. Thus, this study highlighted that the designed IBU-GLU hydrogel could serve as a feasible approach to enhance the release and permeability of IBU for its druggability optimization.

## 1. Introduction

Gels, as distinctive semi-solid dispersions, exhibit the intrinsic viscosity of liquids and the characteristic elasticity of ideal solids [1]. Their inherent three-dimensional (3D) crosslinked networks confer upon them unique rheological behaviors [2,3]. According to the molecular weight of the gelling substances, hydrogel systems are typically categorized into two main types: small-molecule hydrogels and macromolecular hydrogels. For macromolecular hydrogels, the continuous phase is usually constructed via covalent linkages (e.g., disulfide bonds, ester bonds) or non-covalent interactions (e.g., π-π stacking, hydrogen bonds) between natural or synthetic polymers [4,5]. While these macromolecular hydrogels possess high mechanical strength and favorable stability, they often suffer from drawbacks like inadequate biodegradability or suboptimal cytocompatibility. By contrast, small-molecule hydrogels form through the self-assembly behavior of organic molecules (molecular weight < 3000 Da). These molecules typically feature amphiphilic architectures (e.g., cholesterol derivatives) or specific functional moieties (e.g., urea groups, sugar groups), and spontaneously assemble into nanofibers or lamellar structures via non-covalent forces (e.g., π-π stacking, hydrogen bonding), thus building macroscopic gel networks [6].

In contrast to macromolecular hydrogels, small-molecule hydrogels possess inherent theoretical advantages, including thixotropy, thermal reversibility, biodegradability, and biocompatibility. This distinctive set of characteristics endows them with application potential in drug delivery, tissue engineering, and wound healing [7,8]. However, these promising attributes have yet to be fully translated into practical drug development. Certain drugs in crystalline or amorphous forms (e.g., clarithromycin [9], rhein [10], lenvatinib mesylate [11]) tend to aggregate into viscous gel aggregates during manufacturing or dissolution processes, thereby compromising drug performance (e.g., reduced dissolution rate, granulation challenges). Existing studies have demonstrated that single-component hydrogels fail to fulfill the optimal solubilization demands of poorly water-soluble drugs. Herein, whether the development of a binary small-molecule hydrogel system could modulate the physicochemical properties of these drugs was further explored.

Ibuprofen (IBU), as a non-steroidal anti-inflammatory drug, shows antipyretic, analgesic, and anti-inflammatory properties, which is extensively utilized for alleviating mild-to-moderate pain (e.g., headache, arthralgia, and toothache), reducing pyrexia, and mitigating inflammatory reactions [12,13,14]. Clinically, IBU is offered in diverse dosage forms (tablets, capsules, and suspensions). Nevertheless, prolonged oral administration of IBU can induce adverse effects, such as gastrointestinal irritation and renal impairment [15]. Additionally, IBU falls under the BCS Class II ingredient, which is defined by poor aqueous solubility [16]. Its in vivo absorption is constrained by low solubility and dissolution, which motivates ongoing research into formulation advancements. As a supplementary excipient to IBU, N-methyl-D-glucosamine (GLU) is a pharmaceutically feasible solubilizer approved by international regulatory guidelines, and has been utilized in solid oral and parenteral dosage forms to potentiate drug absorption behaviors [17,18,19]. Thus, the current study sought to develop a IBU hydrogel through the incorporation of GLU, with the goal of optimizing the druggability of IBU (i.e., solubility, release profile, and permeability) to broaden its utility in oral and transdermal drug delivery.

## 2. Results and Discussion

### 2.1. Formation and Rheology of IBU-GLU Hydrogel

**Figure 1:**
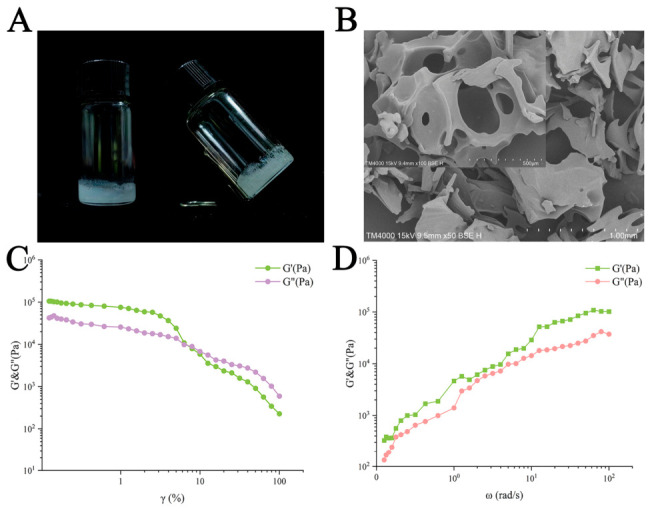
(**A**) Digital photograph and (**B**) SEM morphology of the IBU-GLU hydrogel. (**C**) Strain sweep and (**D**) frequency sweep measurements of the IBU-GLU hydrogel.

Hansen solubility parameter (δ), as an assessment method for characterizing the compatibility of multi-component combinations, has been widely used in ingredient screening and interaction prediction in pharmaceutical formulation design [20]. As seen from Table S1, the δ difference (Δδ) between IBU and GLU was calculated to be 2.40 MPa^1/2^, which is below the critical threshold of 7.0 MPa^1/2^ for phase separation, indicating favorable miscibility between the two components. Then, equimolar IBU and GLU were blended in a small volume of deionized water to facilely construct the IBU-GLU hydrogel. The formed hydrogel exhibited no significant flow behavior when placed in an inclined state (Figure 1A). SEM characterization revealed that the IBU-GLU hydrogel possessed a typical 3D porous network structure formed by the interlaced accumulation of wrinkled sheet-like structures (Figure 1B). Then, the storage stability assays demonstrated that the viscosity of the IBU-GLU hydrogel remained relatively constant for at least one month upon storage at 25 °C, confirming excellent room-temperature stability of the hydrogel system (Table S2).

Rheological analyses provide comprehensive insights into the structural features of gels, covering key properties like flow behavior and rigidity [3]. Strain sweep measurements revealed that both the storage modulus (G′) and loss modulus (G″) of the IBU-GLU hydrogel remained at relatively high levels over the strain range of 0.1% to 10%, with G’ consistently higher than G″ throughout this range (Figure 1C). When the applied strain exceeded 10%, G′ and G″ decreased with increasing deformation, and a modulus crossover (G″ > G′) was observed, indicating the disruption of the hydrogel network. Then, the dynamic frequency sweep test revealed that G′ and G″ of the hydrogel increased synergistically with increasing applied frequency, and G′ remained higher than G″ across the entire frequency range (Figure 1D), exhibiting the distinct elastic-dominant rheological characteristic of the IBU-GLU hydrogel system. Therefore, rheological tests confirmed that the IBU-GLU hydrogel has excellent viscoelastic properties, and G′ remained persistently greater than G″ throughout the linear viscoelastic region (LVR), which was the typical rheological characteristic of a stable gel system [21,22]. The elastic dominance of the hydrogel was derived from the cooperative interactions between the molecular chains of IBU and GLU, and the 3D network structure formed by molecular self-assembly was the structural basis for maintaining stable viscoelasticity. The stable viscoelasticity within a certain strain range could ensure the practical pharmaceutical application of the hydrogel.

### 2.2. Characterization and E_bind_ Calculation of IBU-GLU Hydrogel

**Figure 2:**
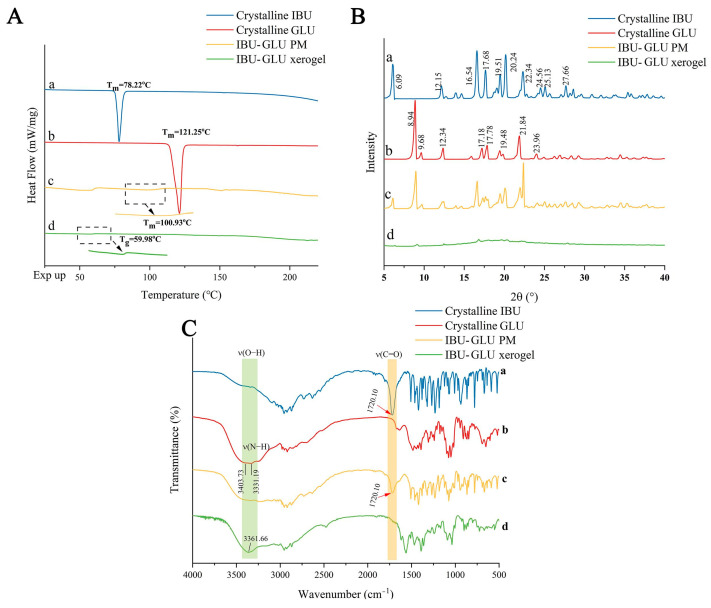
(**A**) DSC thermograms, (**B**) PXRD patterns and (**C**) FTIR spectra of (a) crystalline IBU, (b) crystalline GLU, (c) IBU-GLU PM and (d) IBU-GLU xerogel.

DSC analysis showed that crystalline IBU and GLU exhibited characteristic melting endothermic peaks at 78.22 °C and 121.25 °C, respectively (Figure 2A, a & b), while the melting temperature of the IBU and GLU physical mixture (i.e., IBU-GLU PM) was reduced to 100.93 °C (Figure 2A, c). The IBU-GLU xerogel (obtained by freeze-drying the hydrogel) displayed a single glass transition temperature (T_g_) at 59.98 °C with no obvious melting peaks (Figure 2A, d). PXRD patterns revealed sharp characteristic diffraction peaks for crystalline IBU and GLU (Figure 2B, a & b), and the IBU-GLU PM presented a superposition of the diffraction peaks of the two pure components (Figure 2B, c). In contrast, the IBU-GLU xerogel only exhibited a broad amorphous diffraction halo, indicating a nearly complete amorphous state (Figure 2B, d). FTIR spectroscopy results showed that the C=O stretching vibration peak of IBU at 1720.10 cm^−1^ was absent in the IBU-GLU xerogel, the -OH stretching peak of GLU shifted from 3403.73 cm^−1^ to 3361.66 cm^−1^, and the -NH stretching vibration signal at 3331.19 cm^−1^ disappeared simultaneously (Figure 2C).

DSC and PXRD results collectively confirmed that the IBU-GLU xerogel was in a nearly complete amorphous state, which was a key physicochemical property for improving the solubility of poorly water-soluble drugs such as IBU [23,24]. The disappearance of the crystalline melting peaks of IBU and GLU in the DSC curves (Figure 2A) and their characteristic diffraction peaks in the PXRD patterns (Figure 2B) indicated that the crystal lattice structures of IBU and GLU were destroyed in the hydrogel system. The formation of hydrogen bond complexes between IBU and GLU was the main reason for the amorphization of IBU and GLU (Figure 2C) [25].

Then, the radial distribution function analysis was used to determine the local spatial distribution and then analyzes the structure of hydrogel through the simulation of the preparation process. The result revealed the formation of a strong hydrogen bond between the -OH group (GLU molecule) and the C=O group (IBU molecule) at the distance of 1.6~2.0 Å in the final IBU-GLU-H_2_O cell (Figure S3), which was in agreement with the results of spectral analysis. Furthermore, molecular dynamics simulations were performed to calculate the intermolecular binding energy (E_bind_) of different systems to evaluate intermolecular interactions [23,24,26]. The E_bind_ values of the IBU-GLU-H_2_O, IBU-H_2_O, GLU-H_2_O, and IBU-GLU systems were determined to be 148.069 kcal/mol, 50.985 kcal/mol, 92.446 kcal/mol, and 96.567 kcal/mol, respectively (Table S3). The E_bind_ value of the ternary IBU-GLU-H_2_O gel system was significantly higher than that of the binary systems, indicating the stronger intermolecular interactions within the hydrogel system.

### 2.3. Formation Mechanism of IBU-GLU Hydrogel

The successful formation of the IBU-GLU hydrogel was a synergistic result of favorable component miscibility and robust intermolecular interactions, which aligned with the core principles of small-molecule gel formation [3]. The Δδ between IBU and GLU was far below the 7.0 MPa^1/2^ (Table S1), meaning the component miscibility to facilitate the formation of a homogeneous system [23,24,27]. SEM showed a typical 3D porous network structure (Figure 1B), which stabilized the hydrogel structure by retaining water and resisting dehydration [11]. Furthermore, molecular dynamics simulation results demonstrated that the ternary IBU-GLU-H_2_O system possessed a higher E_bind_ than binary counterparts, indicating that the hydrogel system had stronger intermolecular interactions (Table S3), acting as the driving force for self-assembly and cross-linking [8,28,29]. Then, FTIR spectroscopy further confirmed hydrogen bonding between IBU and GLU as the main non-covalent interaction in the hydrogel (Figure 2C).

**Figure 3:**
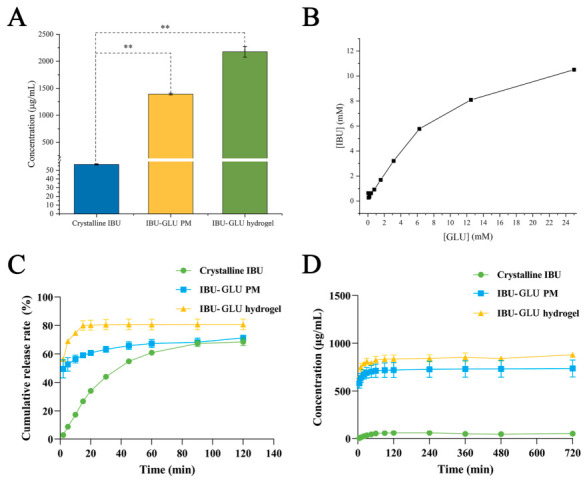
(**A**) Apparent solubility of crystalline IBU, IBU-GLU PM and IBU-GLU hydrogel. (**B**) Phase-solubility diagram of the IBU-GLU system in water. (**C**) cumulative release profiles and (**D**) supersaturated release profiles of crystalline IBU, IBU-GLU PM and IBU-GLU hydrogel. ** *p* < 0.01, compared with crystalline IBU.

Based on the above characterizations of FTIR, SEM and E_bind_, etc., the gelation mechanism of IBU-GLU hydrogel was put forward. From a theoretical standpoint, hydrogel formation is considered the equilibrium outcome of molecular dissolution and aggregation [2,30]. In other words, a hydrogel is formed when both the hydration and aggregation of molecules reach a relatively high level simultaneously. In the initial stage, the IBU molecule and the GLU molecule underwent dissolution in contact with water. According to the phase solubility study, the addition of GLU promoted this dissolution-aggregation equilibrium by increasing the water solubility of IBU (Figure 3B), which was the key to hydrogel formation that a single component (IBU or GLU) could not achieve [11,30]. As the component concentration increased, intermolecular hydrogen bonding interactions induced the dissolved molecules to gradually self-assemble into 3D gel network, thereby forming the IBU-GLU hydrogel. In addition, the excellent storage stability of the hydrogel might be attributed to the synergistic effect of the moisture-locking mechanism of the 3D network and the stable intermolecular hydrogen bond interactions.

### 2.4. Apparent Solubility and Phase Solubility of IBU-GLU Hydrogel

Apparent solubility tests were conducted at 37 °C to evaluate the solubilization capacity of the IBU-GLU hydrogel. Crystalline IBU exhibited an equilibrium solubility as low as 56.65 μg/mL in deionized water. The solubility of IBU in the IBU-GLU PM increased to 1391.34 μg/mL, representing a 24.56-fold improvement compared with crystalline IBU (*p* < 0.01). The solubility of IBU in the IBU-GLU hydrogel reached 2175.38 μg/mL, achieving a significant 38.40-fold enhancement relative to crystalline IBU (*p* < 0.01) (Figure 3A). The phase solubility study was conducted to evaluate the complexation behavior between IBU and GLU in an aqueous environment. The phase solubility diagram of the IBU-GLU system showed that the solubility of IBU increased linearly with increasing GLU concentration in the low-concentration range (0.048~6.25 mM). When the GLU concentration exceeded 6.25 mM, the solubility curve exhibited a non-linear negative deviation, and the phase solubility profile conformed well to the typical 1:1 A_N_-type complexation model (Figure 3B) [30,31].

The 38.40-fold improvement in IBU solubility by the IBU-GLU hydrogel was a comprehensive result involving molecular complexation and amorphization effects (Figure 3A). First, the formation of a 1:1 complex between IBU and GLU was the core molecular mechanism of solubilization during the release process. The phase solubility study confirmed that the IBU-GLU system conformed to the 1:1 A_N_-type complexation model (Figure 3B) [30,31]. The 1:1 A_N_-type complexation model describes the 1:1 molar ratio binding equilibrium between drug and ligand to form a complex (i.e., IBU + GLU ⇄ IBU-GLU complex). The equilibrium association constant was defined as *K*_1:1_ = [IBU-GLU]/[IBU][GLU]. The formation of soluble IBU-GLU complex in the aqueous phase converted the drug from an insoluble crystalline state to a soluble complex state, thus increasing the dissolved amount of IBU in water. When the GLU concentration exceeded 6.25 mM, the non-linear negative deviation of the solubility curve was due to the self-aggregation of GLU molecules at high concentrations, which reduced the effective free GLU concentration in the aqueous solution and thus weakened its complexation capability and solubilization effect [32,33], a phenomenon consistent with the research results of Nicol et al. [31] on cyclodextrin solubilization systems. Secondly, the complete amorphization of IBU in the hydrogel system was also an important structural factor for solubility and release improvement. The disordered molecular packing in the amorphous state eliminated the lattice energy inherent to the crystalline form, making the drug molecules easier to dissociate and dissolve in the aqueous phase [25]. This was consistent with the research results of Tu et al. [16] on the amorphous stabilization of BCS Class II drugs using mesoporous silica, and Han et al. [23] also confirmed that the amorphization of flavonoid components with GLU could significantly improve their aqueous solubility.

### 2.5. In Vitro Release Behavior of IBU from the IBU-GLU Hydrogel

The cumulative release rate of crystalline IBU at 20 min was 34.13% under the sink condition, while that of the IBU-GLU PM reached 60.75%. The IBU-GLU hydrogel exhibited a markedly higher cumulative release rate of 80.28% at 20 min, which was 2.35-fold that of crystalline IBU. At 120 min, the release rate of crystalline IBU increased to 68.56%, and that of the IBU-GLU PM was 71.34%. In contrast, the IBU-GLU hydrogel maintained a high release level of 80.82% at 120 min, i.e., 1.17-fold that of crystalline IBU (Figure 3C). For the release in the non-sink condition, crystalline IBU reached a release plateau at 56.92 μg/mL at 90 min and maintained this level thereafter. In contrast, the IBU-GLU PM and IBU-GLU hydrogel exhibited prominent supersaturated release characteristics, with release concentrations reaching 719.39 μg/mL and 814.10 μg/mL at 90 min (*p* < 0.01, compared to crystalline IBU), respectively, and both systems maintained a high supersaturated state for at least 12 h. In comparison to IBU-GLU PM, IBU-GLU hydrogel exhibited a slightly higher supersaturated release concentration. At 12 h, the release concentration of IBU from the hydrogel was 881.06 μg/mL, which was 16.16-fold that of crystalline IBU (54.51 μg/mL) and 1.19-fold that of the IBU-GLU PM (737.38 μg/mL) (Figure 3D).

The IBU-GLU hydrogel exhibited significantly improved release rates and excellent supersaturated release ability under both sink and non-sink conditions (Figure 3C,D), which is of great significance for the potential in vivo absorption of BCS Class II drugs whose absorption is inherently restricted by poor solubility and slow dissolution rate [23,30]. The high release rate and supersaturation of IBU-GLU hydrogel were mainly attributed to the amorphous state of IBU and the formation of a molecular complex, which reduced the energy barrier and accelerated the dissolution and diffusion of drug molecules [23]. While, the IBU-GLU PM also showed an improved release rate, which was also due to the molecular complexation effect between IBU and GLU in the solution, but its effect was lower than that of the hydrogel system.

The most prominent release characteristic of the IBU-GLU hydrogel was its excellent supersaturated release ability under the non-sink condition, and the hydrogel could maintain a high supersaturated state for at least 12 h, which was significantly different from the “spring-parachute” effect of most supersaturated drug delivery systems [24,34]. The “spring-parachute” effect refers to the rapid increase in drug concentration to a supersaturated state followed by a rapid decrease due to recrystallization, which limits the improvement of in vivo absorption [35]. The IBU-GLU hydrogel avoided the recrystallization of IBU and maintained a stable supersaturated state because the molecular complexation between IBU and GLU could effectively inhibit the nucleation and crystal growth of IBU molecules in the solution. Wei et al. [35] confirmed that the complexation between IBU and nicotinamide could induce a “spring-helicopter” dissolution behavior and inhibit drug recrystallization, and the IBU-GLU hydrogel in this study showed a similar “spring-helicopter” or “spring-plateau” supersaturated release effect. The maintenance of a stable supersaturated state could significantly increase the effective concentration of the drug at the absorption site (gastrointestinal tract or skin), thus increasing the concentration gradient of transmembrane diffusion and ultimately improving the in vivo absorption efficiency of the poorly water-soluble drug.

### 2.6. In Vitro Membrane Permeability of the IBU-GLU Hydrogel

**Figure 4:**
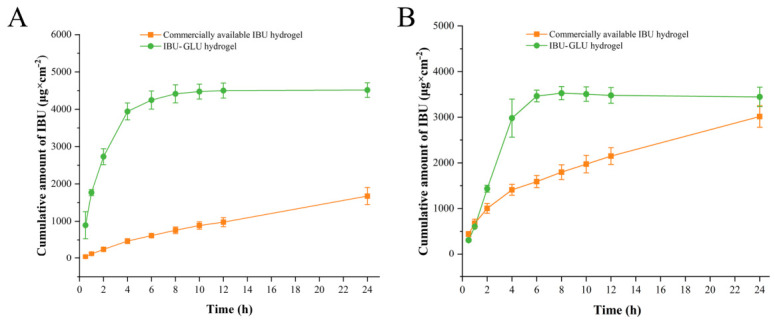
Comparison of cumulative permeation amounts between IBU-GLU hydrogel and commercially available IBU hydrogel through (**A**) PermeaPad^®^ membrane and (**B**) Strat-M^®^ membrane (*n* = 3).

In vitro permeability assays were performed using PermeaPad^®^ membranes (Logan Instruments, Somerset, NJ, USA) (to simulate gastrointestinal epithelial membranes) [30,36] and Strat-M^®^ membranes (Merck Millipore Ltd., Darmstadt, Germany) (to simulate skin membranes) [37,38] with a Franz diffusion cell system, and the performance of the IBU-GLU hydrogel was compared with a commercially available IBU hydrogel (Section S5 in Supplementary Material, trade name: Ibuprofen Gel, Kangzheng Pharmaceutical Co., Ltd., Wuhan, China). The maximum permeation amount of IBU from the IBU-GLU hydrogel through the PermeaPad^®^ membrane was 4516.26 ± 196.53 μg/cm^2^, which was significantly higher than that of the commercial IBU hydrogel (1675.24 ± 228.20 μg/cm^2^) (Figure 4A and Table 1). The transdermal rate (J) of the IBU-GLU hydrogel in the simulated gastrointestinal environment was 22.63 μg·cm^−2^·h^−1^, which was much higher than that of the commercial product (0.67 μg·cm^−2^·h^−1^). For transdermal permeability through the Strat-M^®^ membrane, the maximum permeation amount of the IBU-GLU hydrogel within 10 h was 3527.89 ± 144.93 μg/cm^2^, significantly higher than that of the commercial hydrogel (1973.17 ± 191.97 μg/cm^2^). The J value of the IBU-GLU hydrogel for transdermal permeation was 17.40 μg·cm^−2^·h^−1^, which was significantly higher than that of the commercial hydrogel (0.52 μg·cm^−2^·h^−1^) (*p* < 0.01) (Figure 4B and Table 1). Hence, the IBU-GLU hydrogel showed significantly improved transmembrane permeability in both simulated gastrointestinal and skin environments compared with the commercially available IBU hydrogel, which is the key to optimizing the druggability of IBU and expanding its application in oral and transdermal drug delivery systems (Figure 4). The PermeaPad^®^ and Strat-M^®^ membranes used in the study are well-recognized cell-free permeability models that can accurately simulate the permeability characteristics of gastrointestinal epithelial membranes and skin membranes, respectively (Section S4 in Supplementary Materials), and the test results have good predictability for in vivo permeability [30,36,37,38].

In theory, the release mechanism of macromolecular gels consists of two stages: the initial swelling stage (where solvent molecules penetrate and cause volume expansion) [38,39], and the subsequent dissolution stage (where the movement of macromolecular segments enhances the release of solutes from the polymer network and diffusion into the solution) [40]. The low permeability of the commercial IBU hydrogel was mainly due to its formulation composition. It contained a large number of macromolecular polymer additives (such as carbomer), which formed a dense cross-linked polymer network. This network produced significant spatial steric hindrance, which limited the diffusion of drug molecules through the membrane pores and thus reduced the transmembrane permeability [30,40]. In contrast, the IBU-GLU hydrogel was a small-molecule hydrogel without macromolecular chain cross-linking. The non-covalent hydrogen bonds in the hydrogel could be rapidly dissociated when contacting with the aqueous medium, and the hydrogel network was quickly disintegrated to release free drug molecules, which significantly improved the diffusion efficiency of drug molecules. In addition, the high release rate of the IBU-GLU hydrogel provided a sufficient concentration gradient between the donor and receptor chambers of the Franz diffusion cell, which was the driving force for transmembrane diffusion. The combined effect of the above two factors resulted in the significantly higher transmembrane permeability of the IBU-GLU hydrogel compared with the commercial product. This result is consistent with the conclusion of Furuishi et al. [17] that GLU-based ionic liquids can be used as effective permeation enhancers, and further confirms that GLU could not only improve the solubility of drugs but also enhance their transmembrane permeability, which was a multifunctional pharmaceutical excipient.

### 2.7. Application Prospects and Study Limitations of IBU-GLU Hydrogel

This study successfully prepared a IBU-GLU small-molecule hydrogel via a simple one-step mixing method without complex equipment or harsh reaction conditions. The formed hydrogel significantly improved the solubility, release rate, and membrane permeability of IBU, providing a viable approach for the druggability optimization of BCS Class II poorly water-soluble drugs. At present, the research on pharmaceutical hydrogels predominantly concentrates on macromolecular gels based on natural or synthetic polymers (e.g., starch, cellulose, carbomer) [4,5], while the research on small-molecule drug hydrogels is relatively limited. Most investigations into small-molecule hydrogels focus on solving the problem of gelation-induced performance degradation of single-component drugs [41,42,43], while the binary small-molecule hydrogel system designed in this study actively utilizes small-molecule self-assembly and intermolecular interactions to construct a hydrogel delivery system, which makes up for the deficiency of single-component small-molecule hydrogels in the solubilization of poorly water-soluble drugs.

Moreover, the hydrogel exhibited excellent performance in both simulated gastrointestinal (oral delivery) and skin (transdermal delivery) permeability assays, which could expand the application of IBU dosage forms. For example, the IBU-GLU hydrogel can be formulated into a soft capsule formulation for oral absorption enhancement. In addition, transdermal delivery can avoid the first-pass effect of the liver and reduce the gastrointestinal irritation caused by long-term oral administration, which is of great significance for improving the clinical application safety of IBU. For other poorly water-soluble drugs containing polar groups (e.g., carboxyl, hydroxyl, and amino), this binary small-molecule hydrogel strategy based on GLU could also be used for reference. GLU might form hydrogen bonds and molecular complexation with most of these drugs, thus realizing the optimization of their pharmaceutical properties (solubility, release and permeability). Although the IBU-GLU hydrogel exhibited excellent in vitro pharmaceutical properties, this study still has some limitations that need to be addressed in future research. Firstly, the current research is limited to in vitro experiments, and the in vivo absorption and pharmacodynamic effects of the hydrogel need to be further verified through future animal experiments. In addition, the formulation of the IBU-GLU hydrogel would be further optimized, such as adjusting the molar ratio of IBU and GLU, adding appropriate excipients, to further industrialize the hydrogel product as a modified new drug. In future development, the properties of the hydrogel still need to be further evaluated, such as formulation pH, spread ability, drug-excipient compatibility, and skin irritation safety.

## 3. Conclusions

In the present study, a IBU-GLU composite hydrogel was facilely fabricated through a straightforward mixing approach, where IBU was blended with low-molecular-weight GLU in a trace amount of water. The hydrogel formation was mainly driven by favorable component compatibility and intermolecular interactions dominated by hydrogen bonding. The as-prepared IBU-GLU hydrogel not only remarkably enhanced the apparent solubility and in vitro release behavior of IBU, but also possessed outstanding transmembrane permeation capability. In addition, further investigations, including phase solubility determination, revealed that the complexation between IBU and GLU, combined with the high-energy characteristic of the formed hydrogel, collectively accounted for the solubilization mechanism of the IBU small-molecule hydrogel system. Overall, this study elucidated the formation and solubilization mechanisms of IBU-GLU hydrogel, and presented that such a design strategy could serve as a viable formulation route to broaden the potential application of IBU and even other insoluble ingredients in oral and transdermal delivery systems.

## 4. Materials and Methods

### 4.1. Materials

IBU and GLU were commercially sourced from Sigma-Aldrich Ltd. (Saint Louis, MO, USA) with a purity exceeding 99%. Relevant organic solvents were purchased from Macklin Biochemical Co., Ltd. (Shanghai, China), and phosphoric acid was offered by Merck Co., Ltd. (Rahway, NJ, USA). An ELGA LabWater Purelab Ultra apparatus (Shanghai, China) was utilized to generate purified deionized water.

### 4.2. Preparation of IBU-GLU Hydrogel

A simple one-step blending strategy was used to prepare the IBU-GLU hydrogel. In the preliminary experiment assessment, the influence of the component molar ratios (3:1~1:3) on the formation of the IBU-GLU hydrogel was observed through the photography and polarizing light microscopy, IBU and GLU combination exhibited the best gelation performance at 1:1 molar ratio without crystal residue, indicating that an equimolar proportion of IBU and GLU was sufficient for self-assembly and cross-linking to form the hydrogel system. Then, equimolar IBU and GLU were accurately weighed to a total mass of 1000 mg. The mixed powders were transferred into a vessel and shaken continuously for 15 min to obtain a homogeneous physical mixture, which was designated as IBU-GLU PM. Afterwards, the pre-prepared IBU-GLU PM (1000 mg) were dispensed into individual 10 mL transparent glass vials, supplemented with 200 μL of deionized water, and shaken for another 10 min. Under such conditions, the IBU-GLU mixture self-assembled into a homogeneous IBU-GLU hydrogel system.

### 4.3. Characterization of IBU-GLU Hydrogel

#### 4.3.1. Scanning Electron Microscopy (SEM)

The microscopic morphology of the synthesized IBU-GLU hydrogel was observed by SEM (TM4000, HITACHI Co., Marunouchi, Japan). Prior to the microscopic examination, the hydrogel was subjected to fixation treatment, and a conductive film was formed on its surface through gold plating technology to enhance image resolution and reduce the impact of charge accumulation on imaging.

#### 4.3.2. Rheological Studies

The rheology of IBU-GLU hydrogel was tested using a HAAKE MARS 40 rotational rheometer (Thermo Fisher, Waltham, MA, USA). A parallel plate measuring system with a diameter of 20 mm was employed for all rheological tests, with the vertical gap between the two plates calibrated to a fixed distance of 1.0 mm. The entire testing procedure was conducted at a constant temperature of 25 °C to ensure stable and reproducible experimental data. Two conventional scanning modes were implemented to characterize the viscoelastic behaviors of the hydrogel. Specifically, strain sweep tests were performed at a constant frequency of 1 Hz with the strain varying from 0.1% to 100%, while frequency sweep measurements were carried out under a fixed strain of 3% across a frequency range of 0.1 to 100 rad/s.

#### 4.3.3. Stability Assessment During Storage Process

The obtained IBU-GLU hydrogel was hermetically stored in 10 mL glass vials to prevent solvent loss and exogenous contamination, and was stored under the room temperature condition (25 °C). The storage stability of IBU-GLU hydrogel was investigated to analyze its changes in viscosity through rheological tests on different days.

#### 4.3.4. Differential Scanning Calorimetry (DSC)

The thermal characteristics of the IBU-GLU xerogel were investigated using a DSC 5+ instrument (Mettler Toledo Ltd., Greifensee, Switzerland). The xerogel sample was prepared by subjecting the pre-synthesized IBU-GLU hydrogel to a freeze-drying treatment. For the DSC measurement, roughly 5 mg of the dried IBU-GLU xerogel was precisely weighed and loaded into a standard sealed sample pan. The thermal scanning process was implemented under a continuous nitrogen purge atmosphere, with the sample temperature programmed to rise from 25 °C to 240 °C at a constant heating rate of 10 °C per minute.

#### 4.3.5. Powder X-Ray Diffractometry (PXRD)

The crystalline characteristic of the IBU-GLU xerogel was evaluated via powder X-ray diffraction (PXRD) with a Bruker D8 Advance diffractometer (Bruker Corporation, Karlsruhe, Germany). Before the test commenced, the xerogel sample was flattened and mounted evenly on the sample stage for subsequent scanning. The PXRD patterns were collected over a 2θ range of 5° to 40°, and the step size was set to 0.02° during the entire testing procedure.

#### 4.3.6. Fourier Transform Infrared Spectroscopy (FTIR)

Fourier transform infrared spectroscopy (FTIR) was utilized to analyze the functional group composition of the IBU-GLU xerogel, and relevant spectra were recorded on an INVENIO FTIR spectrometer (Bruker Corporation, Karlsruhe, Germany). For the measurement, the IBU-GLU xerogel was loaded into the sample compartment of the instrument, and spectral scanning was performed over the wavenumber range of 4000 to 500 cm^−1^.

### 4.4. Apparent Solubility of IBU-GLU Hydrogel

An apparent solubility test was conducted to evaluate the solubilization performance of IBU-GLU hydrogel. Firstly, excess samples (including crystalline IBU, IBU-GLU PM, and IBU-GLU hydrogel) were separately placed in 10 mL centrifuge tubes, followed by the addition of 5 mL of deionized water to each tube. The experiment was conducted at 37 °C/200 rpm in a constant temperature shaker. The experiment lasted for 24 h to reach the equilibrium state of solubility (*n* = 3).

The collected supernatants were filtered through a 0.22 μm polyethersulfone (PES) membrane. Subsequently, 1 mL of the obtained filtrate was blended with an equivalent volume of methanol for subsequent determination. The concentration of IBU was determined by high-performance liquid chromatography (HPLC, Nexera LC-40, Shimadzu, Nakagyo-ku, Japan) equipped with a Sharpsil-U C18 chromatographic column (4.6 mm × 250 mm, 5 μm). A mixed solution of acetonitrile and 0.3% phosphoric acid aqueous solution (70:30, *v*/*v*) was adopted as the mobile phase. The HPLC detection conditions were set as follows: a constant flow rate of 1 mL/min, column temperature maintained at 35 °C, and a fixed detection wavelength of 220 nm.

### 4.5. In Vitro Release of IBU-GLU Hydrogel

#### 4.5.1. In Vitro Release Under the Sink Condition

The dissolution apparatus (RCZ-12A, Huanghai Instrument Co., Ltd., Shanghai, China) was used to conduct the cumulative IBU release on crystalline IBU, IBU-GLU PM and IBU-GLU hydrogel (with a dosage equivalent to 10 mg of IBU). The release experiment was conducted under the conditions of 37 °C, 100 rpm paddle speed, and 900 mL deionized water (*n* = 3). At preset time intervals, 2 mL of the solution was sampled, and isothermal fresh medium of equal volume was immediately added to replace the removed portion. The collected sample solutions were filtered through a 0.22 μm PES membrane and diluted with methanol at a 1:1 volume ratio. The IBU concentrations of the processed samples were quantified using the aforementioned HPLC analytical method.

#### 4.5.2. In Vitro Release Under the Non-Sink Condition

The supersaturation release behavior was analyzed for three groups: crystalline IBU, IBU-GLU PM, and IBU-GLU hydrogel. Each group was dosed to be equivalent to 200 mg IBU, and the supersaturation release experiments were implemented in 200 mL of deionized water at 37 °C with a stirring speed of 100 rpm, with three parallel replicates set for each group. Notably, the IBU-GLU hydrogel sample was encapsulated in dialysis bags with a molecular weight cutoff of 14,000 Da prior to the test, which was far higher than the molecular weight of IBU (206.28 Da) and GLU (195.21 Da). The dialysis bag only served to fix the integrity of the hydrogel and avoid sample loss, rather than hindering drug release. 2 mL solution was sampled at the preset time nodes, and isothermal and equal-volume fresh medium was supplemented simultaneously to keep the system volume constant. The collected aliquots were first passed through a 0.22 μm PES filter membrane, and further mixed with methanol at a 1:1 volume ratio. Thereafter, the IBU concentration was quantified under the aforementioned analytical conditions.

### 4.6. Solubilization Mechanism by Phase Solubility Study

Phase solubility measurement was performed to verify whether IBU formed complexes with GLU in an aqueous environment. Excess pure IBU powder was dispensed into separate 10 mL centrifuge tubes, and 5 mL of GLU aqueous solutions with gradient concentrations were added to each tube (*n* = 3). The prepared samples were incubated in a thermostatic shaker under continuous agitation at 37 °C and 200 rpm for 24 h to reach thermodynamic equilibrium. After incubation, 2 mL of the supernatant was collected and clarified using a 0.22 μm PES membrane filter. The concentration of IBU in the obtained filtrate was ultimately determined via the established HPLC detection procedure mentioned above.

### 4.7. In Vitro Permeability of IBU-GLU Hydrogel

A permeability comparison was conducted between the prepared IBU-GLU hydrogel and a commercially available IBU hydrogel (trade name: Ibuprofen Gel, produced by Kangzheng Pharmaceutical Co., Ltd., Wuhan, China). In this study, the PermeaPad^®^ membrane and Strat-M^®^ membrane were used to simulate the membrane behaviors of the skin and gastrointestinal tract, respectively (Section S4 in Supplementary Material). The permeability of the two IBU hydrogels was quantitatively assessed via an intelligent transdermal diffusion instrument (RYJ-12B, Huanghai Instrument Co., Ltd., Shanghai, China). The PermeaPad^®^ membrane, as an artificial cellulose-phospholipid biomimetic membrane, can be used to test the apparent permeability coefficient of drugs [29,30]. Similar to human skin, the Strat-M^®^ membrane is composed of two layers of polyethersulfone and one layer of polyolefin. These polymer layers form a porous structure with transmembrane gradients in terms of pore size and diffusion rate. Furthermore, the porous structure is filled with a proprietary synthetic lipid mixture, endowing the synthetic membrane with skin-like properties [31,32].

In short, the PermeaPad^®^ membrane was securely sandwiched between the donor and receptor compartments of a Franz diffusion cell, and the as-prepared IBU-GLU hydrogel and commercial IBU hydrogel (containing an equivalent of 20 mg IBU) were separately loaded onto the PermeaPad^®^ membrane in the donor compartment (*n* = 3). The receptor compartment was fully filled with 15 mL of pH 6.8 phosphate-buffered solution (PBS) and subjected to continuous magnetic stirring at 350 rpm under a constant temperature of 37 °C. During the experiment, 0.7 mL of the receptor medium was collected at predefined time intervals, and an equal volume of fresh buffer solution was promptly replenished to maintain a constant volume of the receptor system.

Another transdermal permeation test was performed using a Strat-M^®^ membrane, which was horizontally fixed between the donor and receptor chambers of the diffusion cell with its lipid layer facing upward. A pH 7.4 phosphate buffer supplemented with 30% ethanol was used as the receptor medium. All other experimental parameters remained consistent with those adopted in the gastrointestinal permeability test described above. Samples withdrawn from the receptor compartment were filtered through a 0.22 μm PES membrane, and the IBU content was subsequently determined via the aforementioned HPLC analytical system.

The cumulative permeation rate (L_n_) and cumulative permeation amount (Q, μg·cm^−2^) were computed in accordance with Equation (1) and Equation (2), respectively. By plotting L_n_ versus time (t), the permeation profile was obtained, and the slope of the fitted straight line was defined as the transdermal velocity (J).

(1) $$L_{n}=\frac{Q_{n}}{W}\times 100$$

(2) $$Q=\frac{C_{n}\times V_{0}+\sum_{i=1}^{i=n}C_{i}\times V}{S}$$

L_n_: Cumulative transdermal rate at the *n*th time point;

Q_n_: Accumulated amount of IBU passed through the membrane;

W: IBU content in the hydrogel;

C_n_: IBU concentration in the receptor at *n* time point;

C_i_: IBU concentration in the receptor at the *i* time point;

V_0_: Diffusion cell volume;

V: Receptor volume;

S: Franz cell donor area.

## Figures and Tables

**Table 1:** The cumulative permeation rate of IBU-GLU hydrogel and commercially available IBU hydrogel in simulated gastrointestinal and skin environments (*n* = 3).

	Hydrogels	L_n_ (%)	R^2^	J (μg·cm^−2^·h^−1^)
Gastrointestinal permeability	Commercial IBU hydrogel	L_n_ = 0.67t + 0.34	0.971	0.67
	IBU-GLU hydrogel	L_n_ = 22.63(1 − e^−0.47t^)	0.998	22.63 **
Transdermal permeability	Commercial IBU hydrogel	L_n_ = 0.52t + 3.46	0.972	0.52 **
	IBU-GLU hydrogel	L_n_ = 17.40(1 − e^−0.35t^)	0.921	17.40 **

** *p* < 0.01, compared to the commercial IBU hydrogel.

## Data Availability

Data will be made available on request.

## Supplementary Materials

The following supporting information can be downloaded at https://www.mdpi.com/article/10.3390/gels12070577/s1: Table S1: Theoretical δ calculation of IBU and GLU; Table S2: The viscosity values of IBU-GLU hydrogel at 25 °C (Pa·s) (cP ± SD, *n* = 3); Table S3: Binding energy (E_bind_) of the IBU-GLU-H_2_O system at 25 °C; Figure S1: PLM photographs of (a) crystalline IBU and crystalline GLU, as well as (b) IBU-GLU hydrogel stored at 0 day, 7 days and 14 days; Figure S2. The equilibrium structure of IBU-GLU-H_2_O cell after simulated preparation; Figure S3. Radial distribution function analysis of -OH (GLU) and C=O (IBU) in the final IBU-GLU-H_2_O cell.
