# Supplementary material for: Solubility, Release Behavior and Membrane Permeability of a Ibuprofen Hydrogel Co-Assembled with N-Methyl-D-Glucosamine
Source: Gels. 2026 Jun 29;12(7):577. doi: 10.3390/gels12070577 (PMC13409050; doi:10.3390/gels12070577)
Supplement: Supplementary file 1 [file gels-12-00577-s001.zip › gels-4345877-supplementary.pdf]

Solubility, Release Behavior and Membrane Permeability of a Ibuprofen Hydrogel Co-Assembled with N-Methyl-D-Glucosamine

Guoxun Li †, Xinru Lu †, Caijuan Hu †, Jiaxuan Ji, Xiakang Xiong, YuJia Zhang, Zhenwei Ni, Jue Wang, Jiawei Han * and Xiaoqian Liu *

School of Pharmacy & School of Biological and Food Engineering, Changzhou University, Changzhou 213164, China; liguoxun@cczu.edu.cn (G.L.); 14752109014@163.com (X.L.); hcj@cczu.edu.cn (C.H.); jjx1226zzz@163.com (J.J.); 18913431895@163.com (X.X.); yujia_zhang777@163.com (Y.Z.); nizhenwei811@163.com (Z.N.); wangjue@cczu.edu.cn (J.W.)

* Correspondence: hanjiawei329@cczu.edu.cn (J.H.); chmliux@cczu.edu.cn (X.L.); Tel.: +86-159-5197-9228; (J.H.); +86-151-8978-8736 (X.L.)

† These authors contributed equally to this work.

S1. Miscibility analysis of IBU and GLU

The miscibility of IBU and GLU was systematically evaluated by the Hansen solubility parameter (δ) using Molecular Modeling Pro software (Version 6.3.3, Norgwyn Montgomery Software. Inc). The solubility parameter difference (Δδ) between components serves as a critical predictive indicator for assessing miscibility [20]. The δ values could be calculated using the "group contribution method", including dispersion solubility parameter (δ_d), polar solubility parameter (δ_p), and hydrogen bonding solubility parameter (δ_h) (Equation S1). The specific calculations were based on Equation S2, where F_{di}, F_{pi}, and E_{hi} denote the group contribution values for dispersion force, polarity, and hydrogen bonding, respectively, and V represents the group contribution value for molar volume. Theoretically, components are considered to exhibit good miscibility when their Δδ value is below 7.0 $MPa^{1/2}$. Conversely, a Δδ exceeding 10.0 $MPa^{1/2}$ between two components typically indicates immiscibility.

$$\delta^2 = \delta_d^2 + \delta_p^2 + \delta_h^2 \tag{S1}$$

$$\delta_d = \frac{\sum F_{di}}{V},\ \delta_p = \frac{\sqrt{\sum F_{pi}^2}}{V},\ \delta_h = \sqrt{\frac{\sum E_{hi}}{V}} \tag{S2}$$

Table S1. Theoretical δ calculation of IBU and GLU.

Component	δ_d (MPa $^{1/2}$)	δ_p (MPa $^{1/2}$)	δ_h (MPa $^{1/2}$)	δ (MPa $^{1/2}$)	Δδ (MPa $^{1/2}$)
IBU	19.02	8.92	10.53	23.49	2.40
GLU	14.22	10.09	19.15	25.89	

S2. Storage stability

Polarizing light microscopy (PLM, MSD1125BP, Murzider Co. Ltd., China) was applied to distinguish the emergence of crystals from the IBU-GLU hydrogel during storage. In brief, a small amount of sample was placed on a glass slide, which was observed and recorded by PLM under 10 × 4 times magnification. PLM images of samples were shown in Figure S1. Crystalline IBU and crystalline GLU showed obvious birefringence phenomena (Figure S1a). By comparison, the prepared IBU-GLU hydrogel had no birefringence under PLM observation, and such a hydrogel stored at room temperature for 7 days and 14 days did not show any crystalline characteristics either (Figure S1b), suggesting the good physical stability of the formed IBU hydrogel.

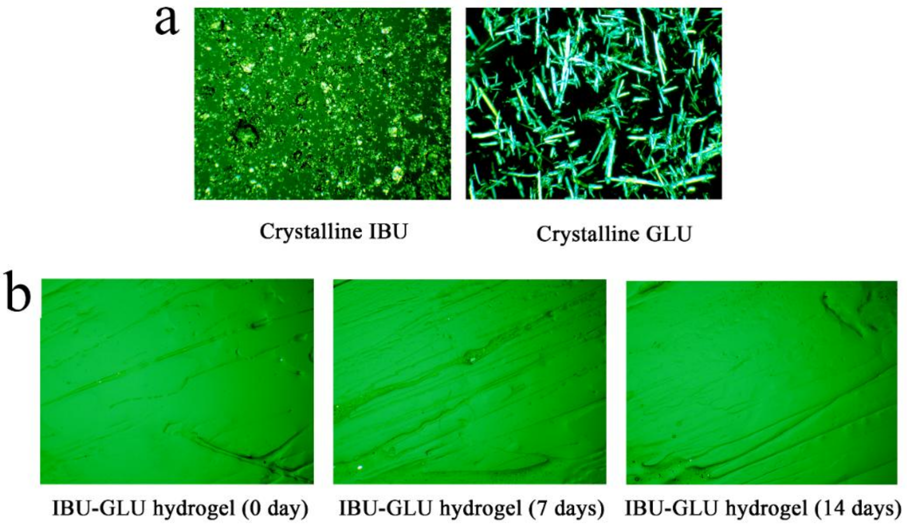

Figure S1. PLM photographs of (a) crystalline IBU and crystalline GLU, as well as (b) IBU-GLU hydrogel stored at 0 day, 7days and 14 days.

Table S2. The viscosity values of IBU-GLU hydrogel at 25 °C (Pa·s) (cP ± SD, n = 3)

Sample	0 day	15 days	30 days
IBU-GLU hydrogel	5569.21 ± 24.26	5520.14 ±36.32	5493.71 ± 54.12

S3. Formation of IBU-GLU hydrogel by molecular dynamics simulation

S3.1. Radial distribution function analysis

(1) Model construction of IBU-GLU-H_2O cell

Step 1: The Forcite module of Materials Studio software (version 2020, BIOVIA) was used for geometric optimization of IBU, GLU and H_2O molecules to minimize their energy [23, 24]. The relevant parameters of MD simulation included Task (Geometry Optimization), Quality (Fine), Forcefield (COMPASS II) and Charges (Forcefield assigned). Besides, Electrostatic and van der Waals were set to Atom based.

Step 2: The optimized molecules of IBU, GLU and H_2O molecules were selected to build the IBU-GLU-H_2O cell by the Amorphous Cell module of Materials Studio software. The molecular ratio of IBU, GLU and H_2O was set to 30:30:134 (equivalent to the amount added in the preparation process). The relevant parameters of MD simulation included Task (Construction), Quality (Fine), Forcefield (COMPASS II), Charges (Forcefield assigned), Electrostatic force (Ewald) and van der Waals (Atom based). In addition, the IBU-GLU-H_2O cell was further optimized to minimize the energy of the system after construction.

(2) Molecular dynamics simulation details

The Forcite module of Materials Studio software was used to simulate the preparation process of the constructed IBU-GLU-H_2O cell. The relevant parameters of MD simulation included Task (Dynamics), Quality (Fine), Ensemble (NPT), Temperature (298 K, i.e., preparation temperature of 25 °C), Presssure (0.0001 GPa),

Total simulation time (200 ps), Time step (1 fs), Number of steps (2×10^5). Other parameters included Thermostat (Andersen), Barostat (Berendsen), Forcefield (COMPASS II), Charges (Forcefield assigned), Electrostatic force (Ewald) and van der Waals (Atom based). After MD simulation, the final IBU-GLU-H_2O cell was obtained for further radial distribution analysis (Figure S2).

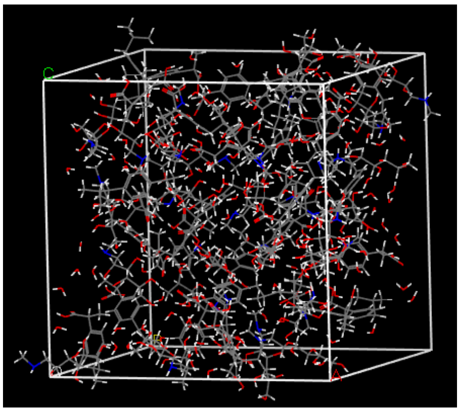

Figure S2. The equilibrium structure of IBU-GLU-H_2O cell after simulated preparation.

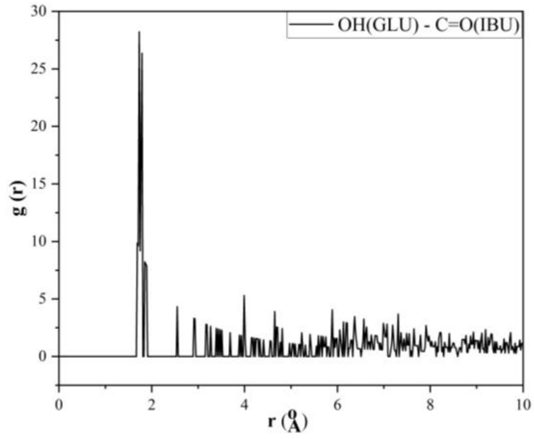

Figure S3. Radial distribution function analysis of -OH (GLU) and C=O (IBU) in the final IBU-GLU-H_2O cell.

S3.2. Binding energy calculation

Small-molecule hydrogels were formed by self-assembly of molecules due to non-covalent interactions or dynamic covalent bonds. Molecular dynamics simulation was used to calculate the binding energy (E_{bind}) between components and further explore the formation mechanism of IBU-GLU hydrogel. The E_{bind} was defined as the intermolecular interaction energy between different components (Equation S3). E_{bind} obtained from molecular dynamics simulation at the molecular level, is frequently used to assess the interactions of two or multiple components, such as in the fields of co-crystal, co-amorphous and amorphous solid dispersion formulations [23, 24, 26]. The Forcite module of Materials Studio software was used to carry out molecular dynamics simulation at 298 K (i.e., the preparation temperature of 25 °C).

$$E_{bind} = -\left[E_{total} - \left(E_{layer(1)} + E_{layer(2)} + \ldots + E_{layer(n)}\right)\right] \tag{S3}$$

E_{bind}: the binding energy of the hydrogel system

E_{total}: the total energy of the entire equilibrium structure

$E_{layer(1)}$, $E_{layer(2)}$ and $E_{layer(n)}$: the total energy of the first, second and n layers

(1) Model construction of IBU-GLU-H_2O cells

Step 1: The Forcite module of Materials Studio software was used for the geometric optimization of IBU, GLU and H_2O molecules to minimize their energy. The relevant parameters of the molecular dynamics simulation included Task (Geometry Optimization), Quality (Fine), Forcefield (COMPASS II) and Charges (Forcefield

assigned). Besides, Electrostatic and van der Waals were set to Atom based.

Step 2: The optimized molecules of IBU, GLU and H_2O were selected to build the IBU-GLU-H_2O cell, IBU-H_2O cell, GLU-H_2O cell, and IBU-GLU cell by the Amorphous Cell module of Materials Studio software. The molecular ratios of IBU, GLU and H_2O were based on the molar mass of the actual preparation. The relevant parameters of molecular dynamics simulation included Task (Construction), Quality (Fine), Forcefield (COMPASS II), Charges (Forcefield assigned), Electrostatic force (Ewald) and van der Waals (Atom based). In addition, these cells were further optimized to minimize the energy of the systems after construction.

(2) Molecular dynamics simulation details

The Forcite module of Materials Studio software was used to simulate the preparation process of the constructed IBU-GLU-H_2O cell, IBU-H_2O cell, GLU-H_2O cell, and IBU-GLU cell. The relevant parameters of molecular dynamics simulation included Task (Dynamics), Quality (Fine), Ensemble (NPT), Temperature (298 K, i.e., preparation temperature of 25 °C), Pressure (0.0001 GPa), Total simulation time (200 ps), Time step (1 fs), Number of steps (2×10^5). Other parameters included Thermostat (Andersen), Barostat (Berendsen), Forcefield (COMPASS II), Charges (Forcefield assigned), Electrostatic force (Ewald) and van der Waals (Atom based). Finally, the equilibrium layered structures of IBU-GLU-H_2O cell, IBU-H_2O cell, GLU-H_2O cell, and IBU-GLU cell could be obtained to calculate their binding energy (E_{bind}) (Table S3).

Table S3. Binding energy (E_{bind}) of the IBU-GLU-H_2O system at 25 °C.

Sample	Total energy	Layer (1)	Layer (2)	Layer (3)	E_{bind}
IBU/GLU/H_2O					
IBU/GLU/H_2O	1218.362	-51.433	2114.36	-696.496	148.069
IBU/H_2O					
IBU/H_2O	-811.913	-49.043	-711.885		50.985
GLU/H_2O					
GLU/H_2O	1286.06	2090.799	-712.293		92.446
IBU/GLU					
IBU/GLU	1978.210	-45.335	2120.112		96.567

S4. Introduction on the PermeaPad® membrane and Strat-M® membrane for permeability evaluation

PermeaPad® membrane, as an artificial cellulose-phospholipid bionic membrane, can be used to test the apparent permeability coefficient (P_{app}) of drugs. PermeaPad® membrane showed a good linear correlation with existing *in vitro* permeation methods in the literature (Caco-2 cell assay or PAMPA data) for high-permeable or low-permeable compounds [30, 36]. In addition, the PermeaPad® membrane can maintain good integrity and surfactant resistance in different pH media and a long-term research environment. Overall, as an innovative bionic membrane, PermeaPad® membrane is a promising scientific tool for quick, economical and reliable evaluation on the gastrointestinal permeability of pharmaceutical formulations.

Furthermore, the Strat-M® artificial skin membrane is a synthetic, non-animal transdermal diffusion test model that can predict the diffusion of substances through

human skin without lot-to-lot differences. Meanwhile, the Strat-M® membrane requires no pretreatment before application, and can be stably stored at room temperature. Like human skin, Strat-M® membrane consists of two layers of polyether sulfone and one layer of polyolefin. These polymer layers form a porous structure with a transmembrane gradient in terms of pore size and diffusivity. Besides, the porous structure is filled with a proprietary mixture of synthetic lipids, giving the synthetic membrane skin-like properties [37, 38].

S5. Introduction of commercially available IBU hydrogel

The commercially available IBU hydrogel (trade name: Ibuprofen Gel, Kangzheng Pharmaceutical Co., Ltd, Hubei, China) presents a colorless and transparent appearance. The product specification is 15 g, and each gram of the product contains 50 mg of IBU component. The pharmaceutical excipients are carbomer, propylene glycol, purified water, and so on. It is used to relieve local soft tissue pain, such as muscle pain, joint pain, and back pain, as well as pain caused by sprains, strains, and overuse injuries. It can also be used for symptomatic treatment of bone and joint conditions.

S6. Statistical analysis

All data were expressed as mean ± standard deviation (SD). Two-way ANOVA was employed to determine significant differences among groups, with the statistical significance cut-off value defined as $p < 0.05$.
